# Polypyrrole Solid-State Supercapacitors Drawn on Paper

**DOI:** 10.3390/nano13233040

**Published:** 2023-11-28

**Authors:** Antonella Arena, Graziella Scandurra, Caterina Branca, Mariangela Ruggeri, Mauro Federico, Valentino Romano, Giovanna D’Angelo, Carmine Ciofi

**Affiliations:** 1 Department of Engineering, University of Messina, 98166 Messina, Italy; arenaa@unime.it (A.A.); carmine.ciofi@unime.it (C.C.); 2 Department of Mathematical and Computational Sciences, Physical Science and Earth Science, University of Messina, 98166 Messina, Italy; caterina.branca@unime.it (C.B.); mariangela.ruggeri@studenti.unime.it (M.R.); mauro.federico@unime.it (M.F.); giovanna.dangelo@unime.it (G.D.); 3 Department of Physics, Politecnico di Milano, Piazza Leonardo da Vinci 32, 20133 Milano, Italy; valentino.romano@polimi.it

**Keywords:** supercapacitor, paper substrates, polypyrrole

## Abstract

Solid-state supercapacitors with areal capacitance in the order of 100 mF⋅cm^−2^ are developed on paper substrates, using eco-friendly, low-cost materials and a simple technology. The electrochemically active material used as the electrode is prepared from a stable water-based ink, obtained by doping commercial polypyrrole (PPY) powder with dodecylbenzene sulfonic acid (DBSA), and characterized by optical and electrical measurements, Raman investigation and Atomic Force Microscopy. The PPY:DBSA ink can be directly applied on paper by means of rechargeable water pens, obtaining, after drying, electrically conducting solid state tracks. The PPY:DBSA layers are then interfaced to one another through a polymer gel based on potassium hydroxide and chitosan, acting both as the ion-conducting medium and as the separator. The areal capacitance of the devices developed by following such a simple rule can be improved when the PPY:DBSA ink is applied in combination with other nanostructured carbon material.

## 1. Introduction

In recent decades, the increasing regard toward the environmental protection and preservation of our ecosystem has produced a strong collective effort, from the manufacturers to the end consumers, to replace, whenever possible, polluting material such as plastic with natural fibers and cellulose. For the same reasons, there has been unprecedented growth in the use of renewable energy sources, known to have a lower impact on the environment when compared to fossil fuels. It is, however, worth underlining how, as far as certain kinds of energy storage systems are concerned, there are still unresolved problems. In addition to safety concerns [1,2,3,4], there is in fact the issue of the disposal of spent devices, often containing toxic elements [5,6,7]. There is also the awareness that the reserve of strategic materials such as lithium and cobalt, often used in batteries, is not limitless and that their extraction from mines comes with high costs in terms of human safety and exploitation [8,9,10]. Finding metal-free, eco-friendly, reliable alternatives to commercially available energy storage devices can therefore be considered amongst the most fascinating scientific challenges for the future.

Among the energy storage systems, supercapacitors (SCs), namely devices whose electrical performances are a bridge between those of capacitors and batteries, are the ideal candidates towards which design innovation can be addressed, to reach a point where these devices can be considered possible replacements for batteries in the near future. Unlike batteries, SCs, which essentially consist of a couple of conducting electrodes interfaced by an ion-conducting medium and a separator, not only already exist in flexible [11], wearable [12], and stretchable forms [13], but they can also be entirely developed using inkjet- and screen-printing technologies [14] suitable for low-cost mass production. Although a sharp demarcation line between the two classes does not exist, according to their operation principle, SCs can be classified as Electrochemical Double-Layer (EDL) supercapacitors or as pseudocapacitors. The capacitance of EDL supercapacitors arises from the electrically driven absorption–desorption of ions at the electrical double layers that form at the interfaces between the electrolyte and the electrodes, the latter usually consisting of nanostructured, carbon-based materials. Compared to EDL SCs, pseudocapacitors have more in common with batteries, as their electrical capacitance originates from redox transitions involving the electrodes, which are therefore required to be electrochemically active. Due to their ability to reversibly commute between an electrically conducting oxidized state and an electrically insulating reduced state, conjugated polymers such as polyaniline [15,16], polypyrrole [17], and polyethelendioxide [18] are among the most commonly used electrode materials in pseudocapacitors. Other common choices of electrodes include a variety of composites containing electrochemically active species, often metal oxides. While capacitance arising from faradaic transition at electrodes is higher compared to that originating from EDL, pseudocapacitors have unresolved cycling stability issues and their electrochemically active electrodes usually have lower conductivity compared to that of the EDL devices. One way to address these problems is to employ electrodes that support both the faradaic and the EDL mechanisms. In such a framework, amongst many other researchers throughout the world, in the past, we developed flexible supercapacitors achieving a few hundred mF⋅cm^−2^ areal capacitance, using gold-coated plastic transparency foils and a nafion membrane containing a lithium compound as substrates and as an ion transporting medium, respectively [19]. As an attempt to reformulate the design excluding lithium, gold, and plastic, here we show how eco-friendly solid-state supercapacitors with lower but acceptable capacitance can be easily developed by using simple technology. The devices are developed by applying, on paper substrates, water-dispersible conducting polymers acting as electrodes and using a chitosan gel containing potassium hydroxide as a spacer and as an ion-conducting medium.

## 2. Materials and Methods

Ionic surfactant dodecylbenzenesulfonic acid (DBSA) 70% in isopropanol, undoped polypyrrole loaded with 20% carbon black, multiwalled carbon nanotubes (CNTs), polyethylene oxide (PEO, average molecular weight 1,000,000), chitosan (medium molecular weight), acetic acid, and potassium hydroxide were purchased from Aldrich (Milan, Italy).

One gram of PPY, which comes as an insoluble insulating powder, was added to DBSA in isopropanol at a ratio of 1:5 in weight, and the ensemble was vigorously mixed to obtain a homogeneous paste. Then, 40 mL of deionized water was added to the mixture, which was ultrasonicated for a few hours. The developed water-based PPY:DBSA dispersion was used to fill rechargeable pens that could be used for months.

CNT dispersions were obtained by vigorously mixing carbon nanotubes and either PEO or DBSA at a ratio of 4:1 by weight until homogeneous pastes were obtained, to which a suitable amount of deionized water was added. The developed pastes, diluted with deionized water (100 mg/50 mL), were used to fill rechargeable pens in order to apply tracks on paper to be used as electrodes.

For comparison purposes, the electrical conductivity of the carbon nanotube-based pastes and inks and the PPY:DBSA inks was tested by applying samples of the material on top of the same kind of alumina substrates having platinum interdigitated electrodes on top.

Chitosan is a biopolymer soluble in dilute aqueous solutions of acetic acid that, due to its functional groups, is not only able to host but is also able to interact with inorganic salts, yielding biodegradable ion-conducting gels [20,21]. The ion conductivity of solid-state membranes derived from chitosan strongly depends on the preparation conditions, for instance on the presence of a plasticizer. Moreover, as aging affects the crosslinking degree of the gel, it has a critical impact on the mechanical stability of the gel-derived membranes. The electrolyte used in this work was prepared by adding 40 mL of deionized water to one gram of chitosan powder, previously wet with 5 mL of acetic acid. After magnetic stirring, KOH, at a 1:0.25 ratio by weight to chitosan, was added to the mixture. After the chitosan addition, the mixture was ultrasonicated for a few hours and stored for a few days before use. The obtained gel could be either applied on the top of the electrodes, or it could be deposited on glasses, resulting in self-standing ion-conducting membranes, once dried and detached from the substrates.

**Figure 1:**
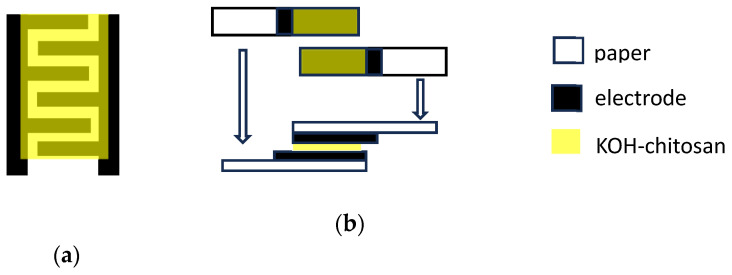
Capacitor structures used in this study: (**a**) interdigitated electrodes are first drawn on paper and the KOH–chitosan gel is drop-deposited on top of the interdigitated section; (**b**) two strips of paper with the electrode and the KOH–chitosan gel are joined together with one strip facing the other.

The capacitors were developed in two different geometries. In the first one, the KOH–chitosan gel was drop-deposited on the gap between interdigitated PPY:DBSA and other kinds of electrodes and applied onto a single sheet of paper by rechargeable pens (Molotov empty marker size 2 mm, and Koh-I-Noor Penna China Professional 04) (Figure 1a). Molotov pens were mainly used to draw geometries by hand, while Koh-I-Noor pens were used in combination with a Graphtec pen plotter MP4300 to demonstrate the possibility of obtaining reproducible and possibly complex electrode geometries. A second geometry, sketched in Figure 1b, was achieved by sandwiching a couple of paper electrodes, obtained by cutting two rectangularly shaped pieces of paper, each having a conducting track of PPY:DBSA and other kinds of electrodes applied on the top, and interposing a KOH–chitosan layer in the middle. To improve the electrical conductivity, samples of the devices have been developed by applying the electrode material on top of layers previously drawn on paper by means of a rechargeable pen filled with commercial carbon-based conducting paste (Electric Paint by Bare Conductive^®^, London, UK, purchased online www.bareconductive.com), diluted with water. For comparison, samples were prepared by using PPY: DBSA, PEO/CNT, and DBSA/CNT as electrode materials.

The morphology of films deposited from PPY:DBSA ink and CNT dispersions was investigated by AFM measurements, which were conducted by means of a Nanosurf FlexAFM equipped with a C3000 controller (Nanosurf AG, Liestal, Switzerland). Optical measurements were performed using an HR4000 Ocean Optics microspectrophotometer. Raman spectra were recorded at room temperature using a LabRam HR800 (Horiba Italia SRL, Rome, Italy) spectrometer with a 532 nm excitation wavelength, an 1800 gr/mm grating, and a liquid nitrogen-cooled CCD camera. The measurements were performed with a 50× objective and a laser power of 1 mW to avoid any heating-induced degradation effects. The electrical measurements were performed at room temperature and at a relative humidity of 58% by means of a Keithley 2400 source meter (Tektronix, Beaverton, OR, USA).

## 3. Results and Discussion

**Figure 2:**
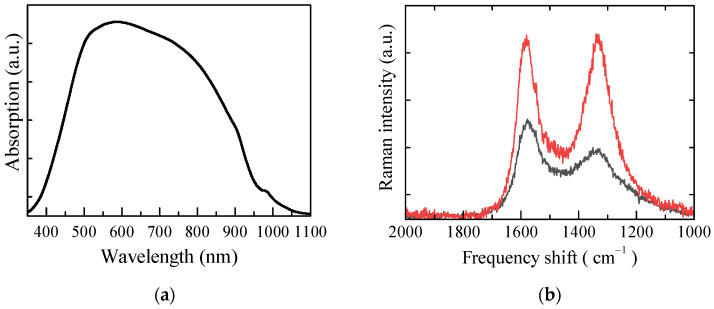
Absorption spectrum of a PPY: DBSA film in the VIS–NIR (**a**); Raman spectrum of PPY (red line) powder compared to PPY:DBSA (black line) (**b**).

Polypyrrole and its derivatives are well-known conjugated polymers that have a variety of technological applications, going from the development of electrochromic windows [22,23,24] to electrochemical gas sensors [25,26,27] and actuators [28], all based on their possibility of existing in an electrically conducting oxidized state and in an electrically insulating reduced state. Doped PPY can be achieved by either chemical or electrochemical polymerization of the pyrrole monomer, carried out in the presence of suitable dopants. The insertion of positive charges provided by the dopant cations, delocalized along the polymer backbone, oxidizes the polymer and produces an increase in electrical conductivity compared to the neutral undoped state. In addition, the presence of the dopant causes a structural rearrangement of the polymer chains and introduces additional electronic states into the bandgap. Such modifications manifest themselves with the appearance of optical absorption bands in the visible–near-infrared region and changes in the infrared and Raman spectra of the doped polymer compared to the undoped state. From the applicational point of view, doped PPY, commercialized as a powder, is virtually insoluble due to strong polymer interchain interactions and poor interactions of the material with the solvents. In a previous paper [19], we succeeded in obtaining stable dispersion of a commercially available doped polypyrrole in water, with the aid of the ionic surfactant DBSA. The excellent quality of the film deposited from such dispersions led to the conclusion that the surfactant, rather than acting as a dopant, not only affects the strong PPY interchain interaction, favoring the dispersion, but forms noncovalent bonds with the polymer backbone. Here, we find that adding suitable amounts of DBSA to commercially available insoluble, undoped polypyrrole powder results in stable inks from which conducting tracks can be deposited on a variety of substrates. Undoubtedly, the surfactant in the present case acts as a dispersing agent for the undoped PPY powder. In addition, since the PPY to be dispersed is undoped, DBSA could also play the role of the doping agent, providing the delocalized cation that oxidizes the polymer. To prove this point, the results of optical spectroscopy in the visible–infrared range and Raman measurements are shown in Figure 2a,b. According to the scientific literature, while polypyrrole in its undoped reduced state has very little optical absorption in the visible region, the material, as the doping level increases, shows absorption peaks arising in the spectral range between 450 nm and the near-infrared range [29]. Such a mechanism is responsible for the polymer’s electrochromic behavior, namely a color change exhibited by the material in response to an externally applied voltage. The presence of a broad band, likely subtending more than one peak, observed in the visible region of the absorption spectrum measured on a thin film deposited on glass from the PPY:DBSA ink (Figure 2a), is, therefore, a demonstration that the surfactant DBSA actually behaves as a dopant for PPY. The change induced in the undoped PPY by the presence of DBSA is also confirmed by the results of Raman measurements. Figure 2b compares the spectrum of the untreated polypyrrole powder with that of PPY:DBSA. The two main peaks positioned at about 1580 cm^−1^ (attributed to C=C in-ring and C-C inter-ring stretching) and 1330 cm^−1^ (attributed to C-C in-ring and anti-symmetric C-N stretching) in the spectrum of the undoped PPY powder are modified in the doped sample, confirming the integration of DBSA in the PPY structure. In particular, the lowest band in doped PPY appears strongly decreased in intensity, slightly shifted in frequency, and enlarged (higher full width at half maximum, FWHM) with respect to the PPY, indicating the structural lattice distortion and higher degree of disorder of the PPY rings [30,31,32].

**Figure 3:**
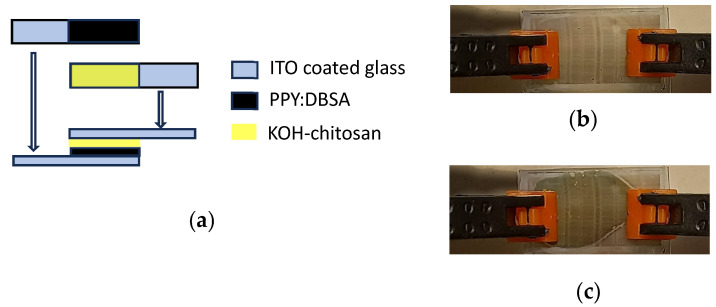
Schematic view of the electrochemical cells used to characterize the PPY:DBSA films (**a**). Photos of a typical electrochemical cell, with the PPY films in the reduced (**b**) and oxidized (**c**) state.

To investigate the electrochemical behavior of the PPY:DBSA ink developed as it was previously described, a simple electrochemical cell has been prepared by interfacing a couple of ITO-coated transparent glasses to each other, one having a thin PPY:DBSA layer applied on the top, and the other having a layer of KOH-doped chitosan on the top. The device, sketched in Figure 3a, exhibits a color change in response to different applied voltages, which are responsible for the change in the oxidation PPY:DBSA film, as shown in the two photographs in Figure 3b,c.

**Figure 4:**
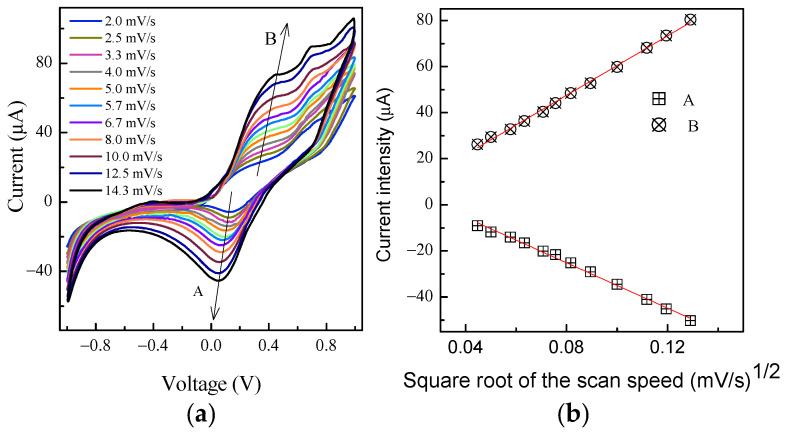
Current–voltage loops measured at different scan speed on a typical device of the kind sketched in Figure 3a (**a**); current intensity measured in correspondence of peak A and peak B, plotted as a function of the scan speed (**b**).

The electrical behavior of the same device has also been investigated by means of current–voltage measurements performed at different scan speeds over a voltage window ranging between −1 V and +1 V, shown in Figure 4.

As reported by Vidanapathirana et al. [33], redox processes occurring in PPY when DBSA is used as a dopant involve the ions provided by the electrolyte interfaced to the doped polymer rather than surfactant ions, which are trapped in the polymer matrix. Accordingly, the current peaks marked by the arrows A and B in the clockwise loops of Figure 4a, the intensities and positions of which change with the scan speed, originate from potassium ions provided by the KOH–chitosan layer, which are either inserted into the polymer matrix when in the reduced state or expelled from it when in the oxidized state. Figure 4b shows that the intensity of peaks marked as A and B in Figure 4a linearly depends on the square root of the scan speed, indicating that the peaks arise from diffusion-limited redox processes at the PPY:DBSA electrodes.

**Figure 5:**
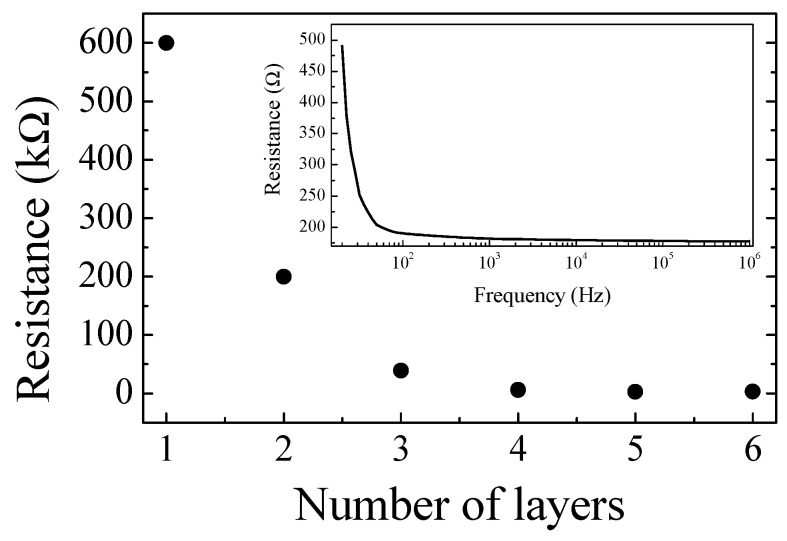
Resistance measured on a track applied on paper as a function of the number of layers of PPY:DBSA ink. The inset shows how the resistance after the application of 6 layers behaves as a function of frequency.

Figure 5 shows how the electrical resistance of a 2 cm long–2 mm wide track applied on paper decreases as the number of layers applied by means of a pen filled with the PPY:DBSA ink increases. It can be noticed that after the first three layers have been applied, the resistance becomes almost constant. The inset shows the real part of the impedance of the same conducting track as a function of frequency (the imaginary part, being two orders of magnitude lower than the real one, is not reported).

**Figure 6:**
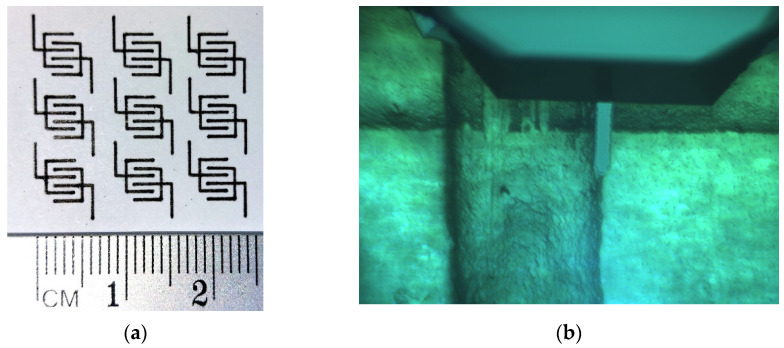
Interdigitated electrodes plotted on paper using the PPY:DBSA ink (**a**); magnified view of a couple of about 0.8 μm thick crossing lines, drawn using the PPY:DBSA ink below the AFM tip (**b**).

In order to test the possibility of using automated drawing systems for producing complex, geometrically consistent, and reproducible patterns, the ink was used to fill up the Koh-I-Noor Penna China Professional 04 that, by means of a proper adapter, can replace a drawing pen for the Graphtec pen plotter. An example of interdigitated test patterns drawn on paper is shown in Figure 6a. AFM is used to measure the step of crossing line patterns drawn on a microscope slide (Figure 6b shows the AFM tip positioned in correspondence with a couple of crossing lines), finding an average thickness of about 0.8 μm for lines obtained after five applications of the ink.

**Figure 7:**
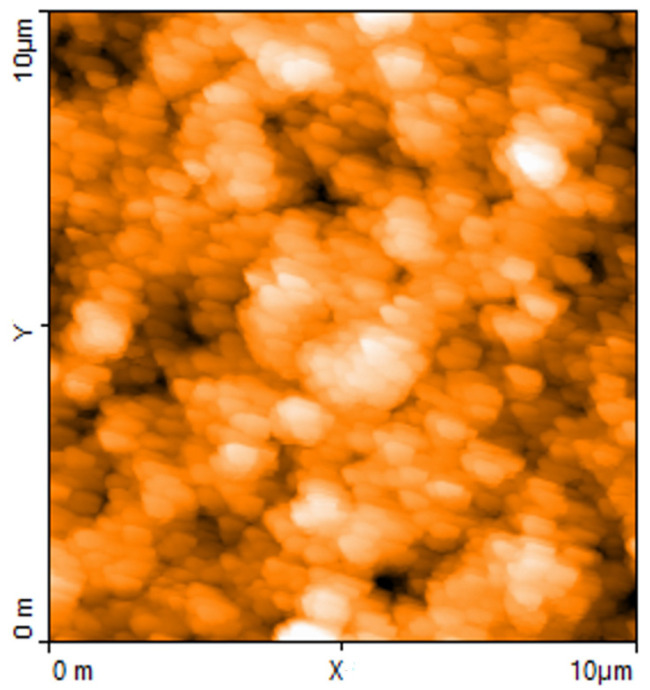
AFM image (area 10 μm × 10 μm) of a typical PPY:DBSA film.

From the morphological point of view, the PPY:DBSA films deposited from the ink have a granular consistency, with hundred-nanometer-size oblong granules. The sample imaged in the AFM picture of Figure 7 is a single track applied onto a silicon substrate by means of a Molotov pen filled with the PPY:DBSA ink. As we found earlier in other mixtures containing the DBSA [19,34], the film appears to have a rather ordered structure, with a certain degree of alignment of the granules along a preferential direction.

**Figure 8:**
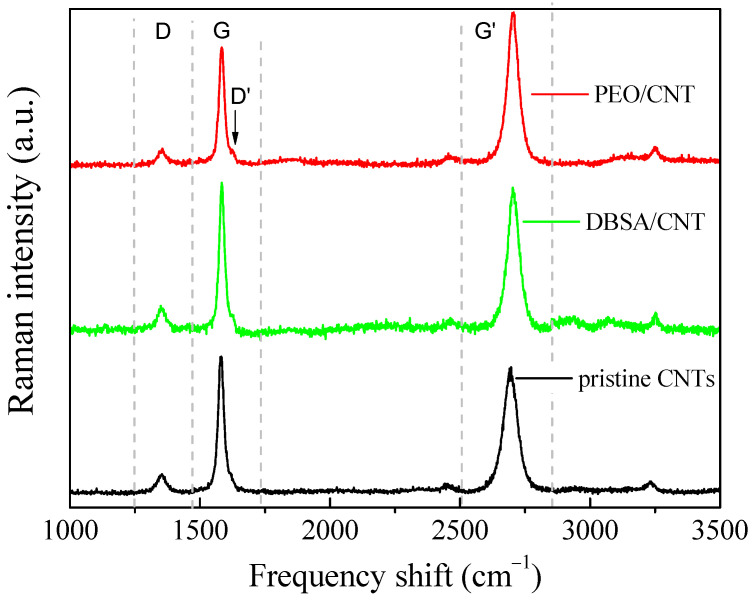
Raman spectra of pristine CNTs, DBSA/CNT, and PEO/CNT hybrid nanocomposites.

As mentioned before, aimed at improving the capacitance of the electrochemically active electrodes, the faradaic contribution provided by the redox transitions can be combined with the good charge transport features and porous nature of nanostructured carbon, which favors the formation of electrical double layers at the interfaces with the electrolyte. For this reason, CNT dispersion in PEO and DBSA has been developed by us as described in a previous section, and characterized by means of Raman and morphological AFM analyses. Raman spectra of the pristine CNTs, DBSA-modified CNTs, and PEO/CNT hybrid nanocomposites are reported and compared in Figure 8. No peaks related to the DBSA or PEO matrix were revealed. Only the MWCNTs contribute to the Raman spectrum, mainly with the characteristic bands of CNTs: the D band (defect) at ~1350 cm^−1^, the G band (graphite band) at ~1580 cm^−1^, and the G’ band (D overtone) at ~2700 cm^−1^.

**Figure 9:**
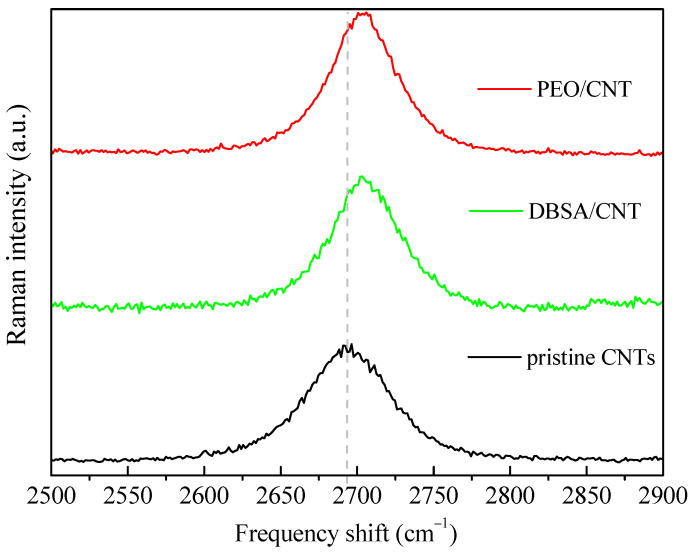
Raman spectra of pristine CNTs, DBSA/CNT, and PEO/CNT hybrid nanocomposites in the spectral region around the G’ band.

The D band defines the in-plane breathing vibration of the aromatic ring. It is activated in the presence of disorder or deviation from the perfect graphitic lattice. The G band arises from the in-plane stretching vibrations of the sp^2^-bonded carbon atoms. Thirdly, the second-order Raman band G’ arises from a double electron–phonon resonance mechanism and can be used to distinguish the number of layers of CNT. At first glance, the spectra are quite similar [35]. But, a careful analysis of the bands and their intensity ratios ID/IG, IG’/IG, and IG’/ID reveals the assessment of the relative influence of DBSO and PEO on the pristine CNTs. For the doped CNT and PEO/CNT hybrid nanocomposites, the G’ band is shifted to higher wavenumber: pristine CNTs: 2694 cm^−1^; DBSA/CNT: 2705 cm^−1^; PEO/CNT: 2703 cm^−1^ (Figure 9). This indicates a charge transfer interaction between the DBSA or PEO and CNTs [36]. Anyway, in all samples, the G’ peak was well fitted by a sharp Lorentzian peak, suggesting the single nature of the CNTs. The intensity ratio of D and G (ID/IG) is commonly used to acquire information on the structure of functionalized CNTs and to estimate the number of defects in the graphitic structure. Indeed, the interaction between the functional groups and the CNT wall is expected to generate a higher number of sp^3^-hybridized carbon with a consequent increase in the ID/IG ratio [37]. Furthermore, the intensity ratio of the G’ and G bands (IG’/IG) and of the G’ and D bands (IG’/ID) was also determined. The IG’/IG ratio indicates the crystalline quality: the higher it is, the more extended the graphitic order is. Finally, the IG’/ID ratio is related to the degree of crystallinity [38].

**Figure 10:**
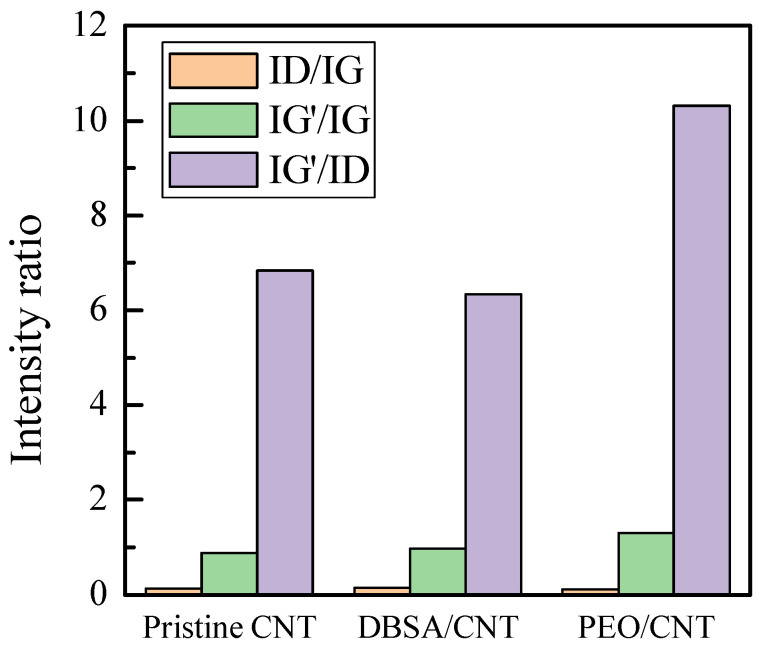
Relative intensity ratios of D/G, G’/G, and G’/D peaks of pristine CNTs, DBSA/CNT, and PEO/CNT hybrid nanocomposites.

The intensity ratios ID/IG, IG’/IG, and IG’/ID are shown in Figure 10. The slight increase in the ID/IG ratio of the doped CNTs (ID/IG = 0.15) compared with that of the pristine CNTs (ID/IG = 0.13) confirms the successful introduction of functional groups onto the CNT surface and that the outer layers of the CNTs were somewhat chemically modified [39]. However, because the ID/IG ratio is small, we can assess that the CNTs have few defects in the modified samples.

The IG’/IG ratio is 0.88, 0.97, and 1.30, whereas the IG’/D ratio is 6.83, 6.33, and 10.32 for the pristine CNTs, modified CNTs, and PEO/CNT hybrid nanocomposites, respectively. The increase in the IG’/IG ratio of the modified CNTs compared with that of the pristine CNTs reveals a lower density of lattice defects in the acid-treated DBSA/CNT and hybridized PEO/CNTs. Furthermore, the hybridized PEO/CNT exhibits a high degree of crystallinity. Interestingly, the G’ peak arises from a two-phonon process; thus, it is expected that its intensity increases as the sample becomes more ordered, enabling the two-phonon coupling process [40]. The higher order in hybridized PEO/CNTs is also confirmed by a decreased full width at half-maximum (FWHM) of the G’ band: pristine CNTs: 65 cm^−1^; DBSA/CNT: 53 cm^−1^; PEO/CNT: 51 cm^−1^.

**Figure 11:**
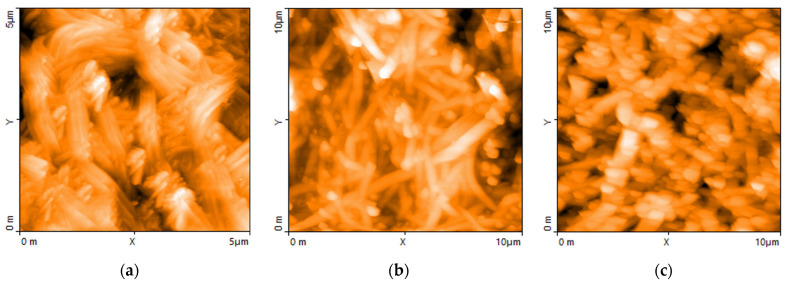
AFM micrograph of pressed CNT powder (**a**) and of CNTs dispersed in PEO (**b**) and in DBSA (**c**).

The difference between PEO’s and DBSA’s carbon nanotube dispersions detected by Raman spectroscopy becomes more apparent when comparing the morphology of a couple of typical samples applied on silicon substrates and investigated by means of AFM measurements. Figure 11a shows the filiform morphology of the pristine carbon nanotubes powder used by us, pressed onto a silicon substrate. Wider filaments of what could be identified as polymer-wrapped nanotubes are recognizable in the AFM image of a film of carbon nanotubes dispersed in PEO deposited on silicon (Figure 11b). On the contrary, the morphology of the carbon nanotubes dispersed in DBSA (Figure 11c), which shows no filaments, resembles that of the PPY:DBSA film shown in Figure 7, confirming the peculiar role of the surfactant when used as a host on the morphologies of carbon material dispersions [19,34].

From the electrical transport point of view, it is found that samples of the pastes containing carbon nanotubes dispersed in PEO and DBSS, applied on alumina substrates with platinum interdigitated electrodes on the top, yielded lower electrical resistance compared to that of the PPY:DBSA sample. Furthermore, compared to that of PPY:DBSA, the real part of the impedance of both the PEO- and DBSA-based pastes showed little frequency dependence in the range between 20 Hz and 1 MHz.

**Figure 12:**
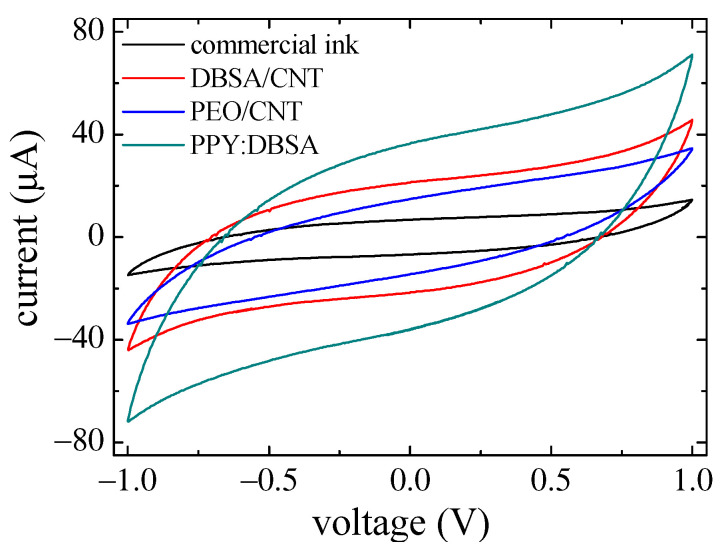
I–V measurements performed on samples having different kind of interdigitated electrodes with the same geometry, applied on paper substrates, with a KOH–chitosan layer on top.

Figure 12 shows the results of current–voltage measurements performed at a scan speed of 10 mV/s on interdigitated electrodes applied on paper, the geometry of which is sketched in Figure 1a, with a KOH–chitosan layer on top. The inner cycle refers to a sample having electrodes deposited using the ink based on commercial conducting mentioned before. The other cycles refer to samples obtained by underlying the commercial ink-conducting tracks with ten layers of PEO/CNT, DBSA/CNT, and o PPY:DBSA, respectively. It can be noticed that better performances in terms of current intensity are achieved by using PPY:DBSA.

Although real devices such as the ones we are investigating are far from behaving as ideal capacitors, a complex model should be developed for interpreting the results of electrical measurements to derive the value of their aptitude to serve as energy storage devices, a rough estimation of the order of magnitude of their capacitance can be derived from time current–voltage loops and from charge–discharge curves. Since the current through an ideal capacitor is given by its capacitance *C* multiplied by the time derivative of the voltage, an estimation *C_es_*_1_ of *C* can be extracted from the IV curve as the ratio between the current at zero voltage (where the current due to the resistive component in parallel is zero) and the absolute value of time rate of voltage change:

(1) $$C_{es1}={\left.\frac{I}{dV/dt}\right|}_{V=0}$$

where *I* is the current and *dV*/*dt* is the scan speed.

Another possibility of the estimation of the capacitance comes from the observation that the finite voltage change Δ*V* over a finite time interval Δ*t* across a capacitor supplied with a constant current *I* is given by the product of the current and the time interval divided by the capacitance itself, and therefore we can obtain the estimate *C_es_*_2_ as:

(2) $$C_{es2}=\frac{I∆t}{∆V}$$

from the observation of charge–discharge curves vs. time at constant current. In the case of an ideal capacitor with no dissipative components, the estimates *C_es_*_1_ and *C_es_*_2_ coincide and are independent of the test conditions. This is not the case in systems such as the ones we are investigating. However, expressions such as the ones in Equations (1) and (2) can still be used to obtain a rough estimate of the capacitance and as an index of comparison among different materials and technologies. Often, in the case in which the active surface *A* of the device can be clearly identified, as in the case of the devices in Figure 1b, estimates are given in terms of the areal capacitance *C_A_* = *C*/*A* and the current density *I*/*A*, rather than in terms of the absolute capacitance and the current, as this provides an information that is independent of the area of the actual device used for testing. In a similar fashion, we can reach approximate estimates for areal energy density *E_A_* and areal power density *P_A_*, per given voltage changes Δ*V* across the capacitor, as:

(3) $$E_{A}=\frac{C_{A}{\left(∆V\right)}^{2}}{2}\;;P_{A}=\frac{E_{A}}{∆t}\;\mathrm{where}\;C_{A}=\frac{I∆t}{A∆V}$$

Tests for estimating capacitance and areal capacitance have been performed on devices built according to both the geometries described in Figure 1a,b.

**Figure 13:**
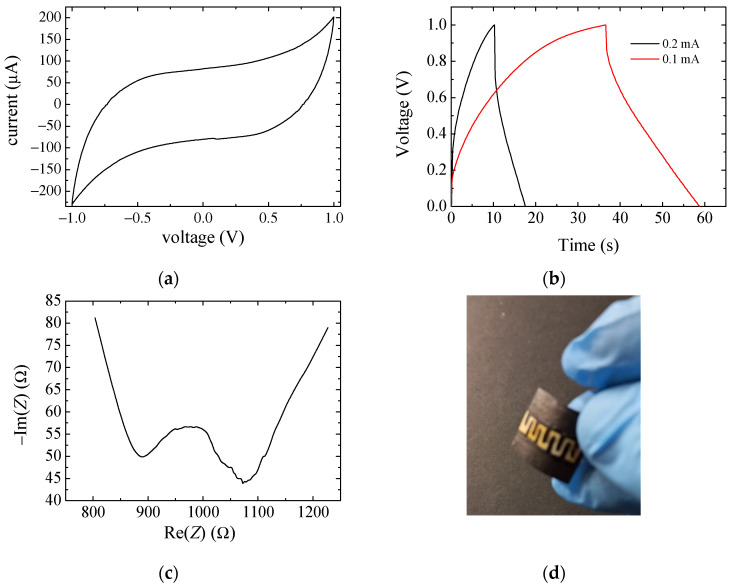
Current–voltage loop (**a**), voltage charge–discharge curve (**b**), frequency-dependent impedance (**c**) and photograph (**d**) of a typical device on paper, with a mixture of the PPY:DBSA and DBSA/CNT inks as electrodes and a KOH–chitosan layer on the top.

The current–voltage loop of Figure 13a refers to a scan performed at 10 mV/s on the sample shown in the photograph in Figure 13d. The device uses as electrodes a mixture of the PPY:DBSA and DBSA/CNT inks, applied on the underlying interdigitated tracks (geometry in Figure 1a). Figure 13b reports the voltage across the sample when charged and discharged at two different levels of constant current. In the case of this type of device, an effective active area cannot be easily defined and therefore we used Equations (1) and (2) for capacitance estimation from Figure 13a and Figure 13b, respectively. From Figure 13a, using Equation (1), we obtain *C_es_*_1_ = 6.5 mF; from Figure 12b, using the time interval required to reach 1 V starting from 0 V, we obtain values for *C_es_*_2_ between 2 and 4 mF, depending on the selected curve (that is, the value of the charging current). As expected, the estimates are different, although we obtain values of capacitance that are in the same order of magnitude, and this confirms that any of the methods discussed above, while not accurate, is useful in providing a rough indication of the charge storage capabilities of the system. Figure 13c shows the opposite of the imaginary part of the impedance, versus the real one, measured between 20 Hz and 1 MHz. From this graph, we can notice the rather high value of the resistive component with respect to the imaginary one, and this is, among other possible causes, the result of the large spacing between the electrodes. It is therefore reasonable to assume that with an optimized design, this aspect can be improved.

**Figure 14:**
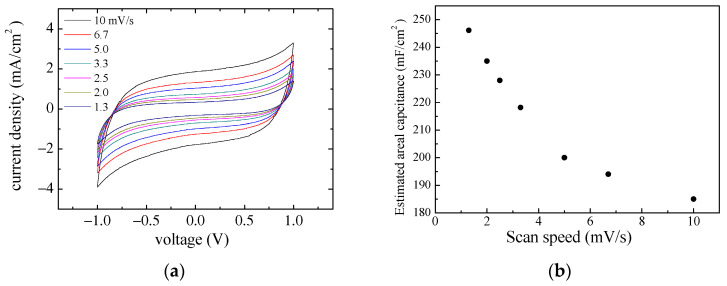
Current–voltage measurements performed at different scan speeds on a sample consisting of a couple of rectangularly shaped pieces of paper, each having a mixture of PPY:DBSA and DBSA/CNT applied on the top, interfaced through a KOH–chitosan layer (**a**); areal capacitance estimated from the current–voltage loops, as a function of the scan speed (**b**).

**Figure 15:**
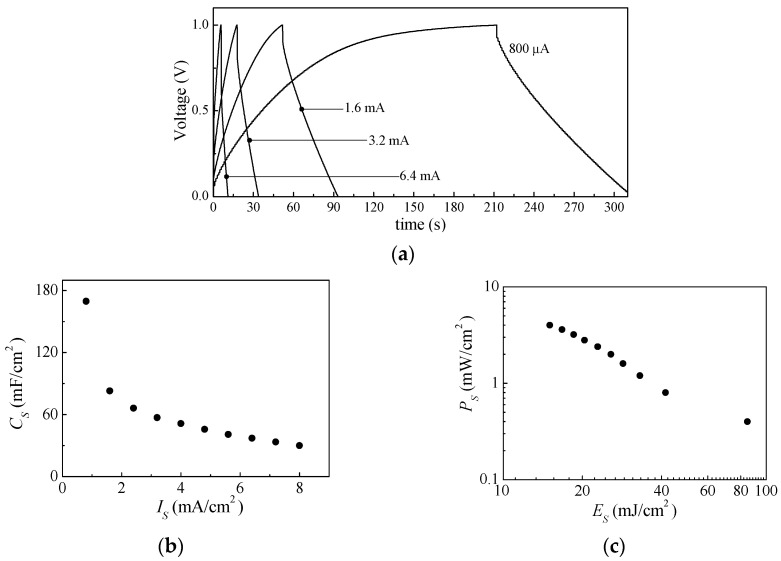
Voltage charge–discharge curves measured on the same device to which Figure 13 refers to (**a**); areal capacitance estimated from the charge–discharge curves, as a function of current density (**b**); power density versus energy density (**c**).

**Figure 16:**
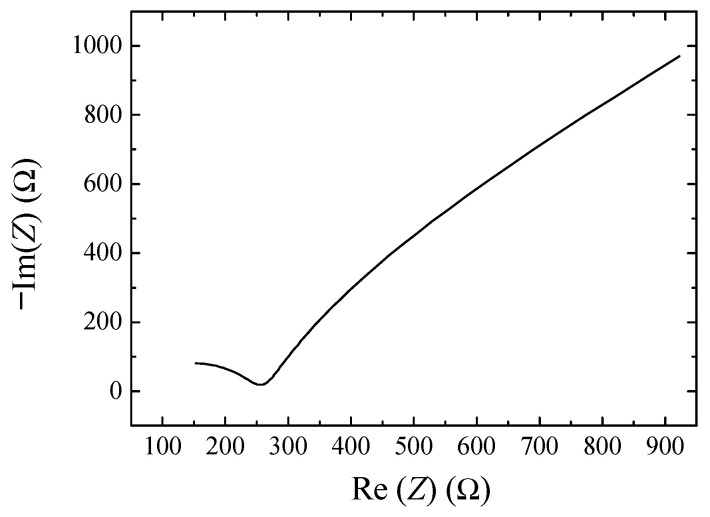
Cole–Cole plot of the same device to which Figure 13 and Figure 14 refer to.

Current–voltage, charge–discharge, and impedance measurements were also performed on a typical device with the geometry in Figure 1b, with electrodes obtained from a mixture of the PPY:DBSA and DBSA/CNT inks. The results are reported in Figure 14, Figure 15 and Figure 16. In particular, Figure 14a shows how the steady state loops of current density versus voltage enlarges as the time rate of voltage change increases. The values of the capacitance per unit area estimated for each loop, and reported as a function of the scan speed, are shown in Figure 14b. Figure 15a shows examples of the charge–discharge voltage measured at different current densities. The areal capacitance estimated from the data in Figure 14a is plotted as a function of the current density in Figure 15b. The obtained values, in the range from 40 to 170 mF/cm^2^ for test current densities ranging from 6.4 down to 0.8 mA/cm^2^, are in the same order of magnitude as those obtained from the current–voltage measurements shown in Figure 14b. The performance of the device under tests in terms of specific energy and specific power, derived from the data of Figure 15a using Equation (3), are summarized in the Ragone plot of Figure 15c. Figure 16 shows the opposite of the imaginary part of the impedance, versus the real one, measured between 20 Hz and 1 MHz. Also, in this case, the real part of the impedance is larger than the imaginary part. In this case, improvement can be possibly obtained by careful optimization of the ink composition.

**Figure 17:**
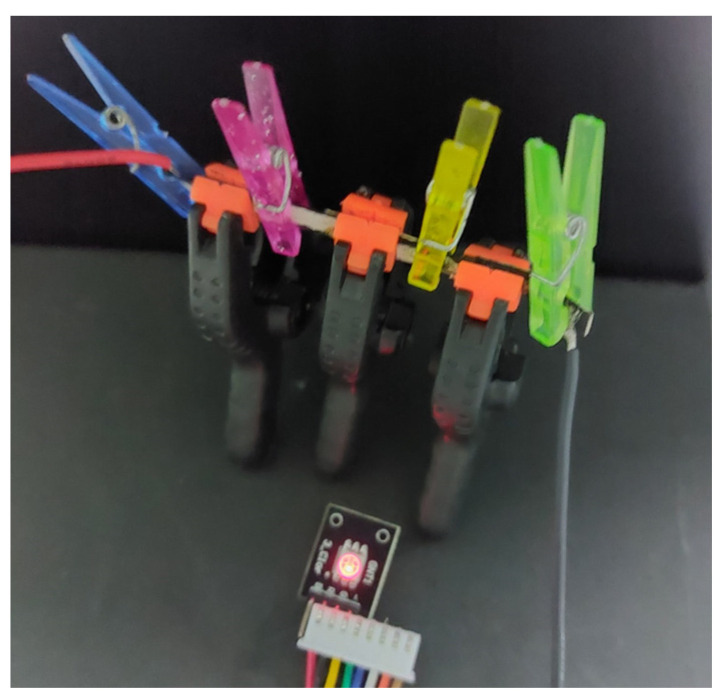
Snapshot of an LED being supplied by three paper devices connected in series. The series of devices was previously charged to a 4 V; the LED, with a 1 kΩ resistance in series, stays on for a few seconds after the supply is removed.

As a demonstration of the charge storage capacity of these devices, Figure 17 shows three of them connected in series (each of which having an active area of 10 mm^2^), while maintaining an LED “on” for a few seconds after being initially charged to 4 V.

**Figure 18:**
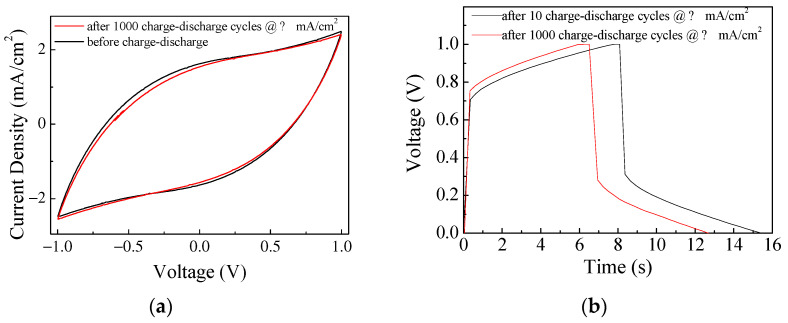
Steady state current–voltage loop measured on a typical device with geometry as described in Figure 1b and PPY:DBSA electrodes, before and after stress (**a**); charge–discharge curve measured at constant current density on the same device, before and after stress (**b**).

As far as stability is concerned, all the developed devices, when stored in air at room temperature, do not show significant changes in the current–voltage loops and in the charge–discharge behavior, even after months. As the capacitance of conjugated polymer electrodes arises from electrically driven redox transitions involving intercalation and de-intercalation of mobile ions into the polymer, such kinds of electrodes are usually the most susceptible to degradation under stress conditions. Figure 18a,b compare the results of current–voltage measurements and charge–discharge measurements, performed before and after 1000 subsequent charge–discharge cycles on the same cell, with the geometry described in Figure 1b and using only PPY:DBSA as the electrode material. It can be noticed that while the IV curve seems to be slightly affected by the stress, the charge–discharge time undergoes a 20% decrease, denoting a significant degradation of the electrode.

## 4. Conclusions

The experimental results presented and discussed here demonstrate that it is possible to develop devices on paper substrates with good specific capacitance by using commercially available, eco-friendly materials such as inks and with the aid of simple and low-cost deposition techniques. The areal capacitances that have been obtained are in the range of 100 mF/cm^2^ (83 mF/cm^2^ at 1.6 mA/cm^2^) with specific powers that can be in excess of 1 mW/cm^2^. However, the performances of capacitors achieved using the approach we propose are, at present, limited by a rather large series resistance. This resistance can be, in principle, reduced by improving the conductivity of the electrodes and the electrolyte by optimizing their composition and processing, and/or by optimizing the geometry of the device by, depending on the configuration, reducing the spacing between interdigitated electrodes or increasing the overlapping surface. The performances obtained so far are encouraging, especially because we employed carbon-based electrodes without any toxic, rare, or precious chemical element or component.

## Data Availability

The data can be requested from the authors.
